# Advances in Biofabrication for Tissue Engineering and Regenerative Medicine Applications

**DOI:** 10.3390/polym13091522

**Published:** 2021-05-09

**Authors:** Marco Domingos, Sam Moxon

**Affiliations:** 1 Department of Mechanical, Aerospace and Civil Engineering, School of Engineering, Faculty of Science and Engineering & Henry Royce Insititute, University of Manchester, Manchester M13 9PL, UK; 2 Division of Cell Matrix Biology & Regenerative Medicine, School of Biological Sciences, Faculty of Biology, Medicine and Health, University of Manchester, Manchester M13 9PL, UK

Biofabrication strategies continue to gain considerable interest in the efforts to develop methods for better replicating in vitro models of human tissues. With applications in drug screening, disease modelling and tissue regeneration it is becoming increasingly clear that techniques like 3D bioprinting and other advanced manufacturing technologies have a very important role to play in the revolution of personalised medicine. Nowhere is this more evident than in the predictions that the market for 3D bioprinting alone will eclipse $1.6 billion within the next three years.

In acknowledgement of this continually evolving research strategy, a Special Issue focussing on some of the latest developments in new biofabrication strategies was launched in 2021. Consisting of five studies, the issue highlights new and exciting developments in the advancement of biofabrication for tissue engineering and regenerative medicine applications.

Sanz-Garcia et al. present a study entitled “A Versatile Open-Source Printhead for Low-Cost 3D Microextrusion-Based Bioprinting” [1]. In this development, the authors showcased a new print head design that can allow many standard desktop 3D printers to be transformed into 3D bioprinters. The head is low cost and easy to build and has the potential to vastly improve the accessibility of 3D bioprinters. Adapting a 3D printer with their bespoke design allowed the printing of multiple bioinks and facilitated the extrusion of cell-based constructs without compromising cell viability. Moreover, open-sourcing of their print head will allow other researchers to apply it to their 3D printing devices. 

In “Towards 3D Multi-Layer Scaffolds for Periodontal Tissue Engineering Applications: Addressing Manufacturing and Architectural Challenges” [2], Porta et al. present a porous poly(ε-caprolactone) (PCL) and Sr-doped nano hydroxyapatite (Sr-nHA) with a multilayer structure. The scaffold was produced via a single-step additive manufacturing process with intended applications in the regeneration of periodontal tissue. The generated construct showed viability for the intended application on multiple fronts. Physiochemical and mechanical testing revealed suitability for periodontal regeneration and human osteosarcoma cells exhibited biocompatibility with the scaffold.

In a third study, Cakmak et al. [3] present a 3D printed composite scaffold comprised of polycaprolactone, gelatin, bacterial cellulose and hydroxyapatite for bone tissue engineering. The authors reported a pore size that is ideal for use in bone tissue engineering applications. Additionally, the presence of hydroxyapatite and cellulose within the scaffold facilitated cell attachment and proliferation and the ability to 3D print the composite could facilitate the generation of personalised scaffolds for bone regeneration.

Another study entitled “Topography-Mediated Myotube and Endothelial Alignment, Differentiation, and Extracellular Matrix Organization for Skeletal Muscle Engineering” [4] presents a method for directing endothelial cell behaviour via topographical cues. A PDMS-based topography was applied to investigate the influence of pre-aligned myotubes on the network formation of microvascular endothelial cells. Alignment of the myotubes resulted in the formation of a laminin/collagen fibre network and stimulated the early stages of endothelial network formation.

In the final study of this Special Issue, the use of a new bioprintable poly(ester urea) (PEU) material as an alternative to polycaprolactone for the generation of an in vitro model of early chondrogenesis is presented [5]. The PEU polymer was successfully printed into 3D scaffolds with defined filament diameters and pore sizes. Human chondrocytes cultured on the scaffolds exhibited higher cell viability and improved chondrogenic phenotype than observed on PCL scaffolds highlighting the potential to apply PEU in cartilage tissue engineering applications.
